# Is PREHAB in Pelvic Floor Surgery Needed? A Topical Review

**DOI:** 10.3390/medicina56110593

**Published:** 2020-11-06

**Authors:** Jacek K. Szymański, Małgorzata Starzec-Proserpio, Aneta Słabuszewska-Jóźwiak, Grzegorz Jakiel

**Affiliations:** 1 1st Department of Obstetrics and Gynecology, Centre of Postgraduate Medical Education, Żelazna 90 str., 00-004 Warsaw, Poland; jkszymanski2@gmail.com (J.K.S.); anetaslabuszewska@gmail.com (A.S.-J.); grzegorz.jakiel1@o2.pl (G.J.); 2 Department of Gynecology, St. Sophia Hospital, Żelazna Medical Center, Żelazna 90 str., 00-004 Warsaw, Poland

**Keywords:** pelvic floor muscle training, stress urinary incontinence, pelvic organ prolapse, prehabilitation, menopause

## Abstract

Pelvic organ prolapse and urinary incontinence affect approximately 6–11% and 6–40% of women, respectively. These pathologies could result from a weakness of pelvic floor muscles (PFM) caused by previous deliveries, aging or surgery. It seems reasonable that improving PFM efficacy should positively impact both pelvic floor therapy and surgical outcomes. Nonetheless, the existing data are inconclusive and do not clearly support the positive impact of preoperative pelvic floor muscle training on the improvement of surgical results. The restoration of deteriorated PFM function still constitutes a challenge. Thus, further well-designed prospective studies are warranted to answer the question of whether preoperative PFM training could optimize surgical outcomes and if therapeutic actions should focus on building muscle strength or rather on enhancing muscle performance.

## 1. Introduction

Prehabilitation (PREHAB) is defined as the process of improving the functional capacity of an individual before a surgical procedure in order to reduce potential complications and enhance surgical success [1]. Different forms of prehab can be distinguished: from complex training containing diverse sensorimotor and strengthening exercises [2] to preoperative pain neuroscience education [3]. Pelvic floor surgery remains challenging. The success rate of various procedures ranges from 40 to 100%. Many factors contribute to obtaining an optimal surgical result. Adequate individualized qualification, preoperative preparation, surgical skills and course of postoperative healing are crucial. Each of these factors could be modified and improved; however, it seems that the proper preoperative preparation of the patient constitutes one of the key points of surgical success. Most of the pathologies of the pelvic floor ensue, in general, from the weakness of the pelvic floor muscles (PFM) resulting from previous deliveries, surgery or aging. PFM training is recommended as the initial therapy for stress, urge or mixed incontinence in women of any age [4]. It seems rational that improving the function of the PFM should positively influence other nonconservative treatments, including the outcomes of surgical procedures. In the literature, there is a paucity of trials contributing to preoperative PFM training in women. The benefits of six-month pelvic floor muscle training in improving symptoms and anatomical relationships at short-term follow-up were confirmed in the Cochrane review on the conservative treatment and prevention of pelvic organ prolapse in women. The authors highlighted the lack of medium- and long-term observations as well as the need for trials combining PFM training and surgery [5]. Surgery remains the golden standard for the management of severe pelvic organ prolapse in women who failed conservative therapy, although the combination of conservative and surgical treatment could be effective for enhancing surgical results. This review aims to investigate the existing evidence regarding PREHAB in pelvic floor surgical management. For the purpose of this review, we have focused on different forms of PREHAB that can be implemented in the urogynecological field. Therefore, we included the articles that investigated the PFM training prior to surgery as well as the studies that assessed the educational components or modifications of daily living activities. Due to the restricted amount of evidence for PREHAB among females, we also looked at research that implemented pelvic floor therapy and perioperative assessment as a part of surgical treatment.

## 2. Discussion

### 2.1. Perioperative Physiotherapy in Female Pelvic Floor Surgery

#### 2.1.1. Perioperative Intervention in Pelvic Organ Prolapse and/or Stress Urinary Incontinence Surgery

Jarvis et al. [6] analyzed the synergistic potential of preoperative physiotherapy and surgery in women with pelvic organ prolapse and stress urinary incontinence. This randomized controlled trial included 60 women, of whom 30 underwent preoperative physiotherapy and 30 were treated only surgically. Women in the study group received an individually selected set of four pelvic floor muscle exercises, which they were to perform daily. Particular attention was paid to the correct execution of the Knack maneuver, which consists of the rapid maximal contraction of the pelvic floor muscles in order to protect the pelvic floor against a sudden increase in intra-abdominal pressure [7]. Additionally, the participants of the treated group were taught the correct defecation and voiding techniques to reduce the need to tighten the abdominal muscles. There were no significant differences between the groups in the reduction of stress urine leakage in the paper towel test. However, the groups differed in their results for urinary symptom-specific health and the quality of life questionnaire, the mean maximum squeeze and the mean difference in daily frequency, favoring the pelvic floor PREHAB group.

#### 2.1.2. Perioperative Intervention in Pelvic Organ Prolapse

The influence of preoperative muscle performance on surgical outcomes and the effect of preoperative PFM training is still controversial, and clinical trials remain inconclusive [8]. The systematic review from the year 2016 also does not give any clear recommendations. Primary outcomes were defined as prolapse symptoms and prolapse-specific quality of life. Secondary outcomes included pelvic floor muscle function, the degree of prolapse, urinary and bowel functions, the activity scale, PFM training adherence and adverse effects. An analysis of five randomized controlled trials (RCTs) including 591 patients showed no improvement in primary and secondary outcomes in women undergoing surgery for genital prolapse combined with PFM training compared to surgery-only groups. Although the authors did not find evidence to support adding preoperative PFM training to surgery, they indicated the need for further research to evaluate the potential benefits from preoperative PFM training and to establish an optimal PFM training regimen using sufficiently long-term follow-ups [9].

#### 2.1.3. Perioperative Intervention in Mixed Urinary Incontinence

The recently published “The Effects of Surgical Treatment Enhanced With Exercise for Mixed Urinary Incontinence” (ESTEEM) study comparing the effect of behavioral training and PFM training associated with surgery vs. surgery alone among women with mixed urinary incontinence revealed only a small statistically significant difference in urinary incontinence symptoms at the 12-month follow-up. This difference did not reach the threshold for clinical importance. The study comprised 416 women with bothersome mixed urinary incontinence who were randomized to sling-only surgery or sling combined with pelvic floor muscle training. The primary outcomes were established as a change in symptoms at one year, based on the long form of the Urogenital Distress Inventory (UDI). The secondary outcomes were determined as a change in UDI-stress and UDI-irritative subscale scores between the groups at 12 months. Although the difference between the groups did not reach the clinical importance attributed to primary and secondary outcomes (adjusted mean change of −128 points vs. −114 points; the model-estimated between-group difference of −13.4 points; 95 confidence interval (CI): −25.9 to −1.0; *p* = 0.04), a statistically significant difference was found in exploratory outcomes including the three-day bladder diary and the incontinence-specific quality of life. Bladder diary results favored the combined group, and this group demonstrated a significantly greater improvement in Incontinence Impact Questionnaire scores. At 12 months, the likelihood of additional treatment for lower urinary tract symptoms in the combined group was significantly lower than in the sling-only group (8.5% vs. 15.7%, odds ratio (OR): 0.47; 95% CI: 0.26–0.85, *p* = 0.008) [10]. Further details about the above-mentioned study are presented in Table 1.

### 2.2. Prehab in Male Pelvic Floor Surgery

The PREHAB concept has been studied much more extensively in males. Several trials were conducted on men who underwent prostatectomy and received PFM training before surgery. Ocampo-Truijllo et al. [11] investigated the effectiveness of preoperative PFM training on histomorphometry, muscle function, urinary incontinence and health-related quality of life in men who were scheduled for radical prostatectomy. The study involved 16 men who were randomized into two groups. The experimental arm subjects received a supervised PFM four-week training regimen, three times a day and one month before surgery. During the surgical procedure, samples of the external urethral sphincter were collected for histomorphometric analysis. The study revealed that preoperative pelvic floor muscle training induces histological changes in the muscles of the pelvis. The cross-sectional area of the muscle fibers was increased in subjects who underwent pelvic floor muscle training (PFMT) (1.313 ± 1.075 μm^2^ vs. 1.056 ± 844 μm^2^, *p* = 0.03). Moreover, this group presented a significantly higher-pressure contraction of the levator ani. (F = 9.188; *p* = 0.010). After removing the catheter from the bladder, 62% of the participants in the experimental group showed no incontinence and 75% of the subjects in this group did not require any pads compared to 37% and 25% in the control group, respectively. However, the difference did not reach statistical significance. Furthermore, no significant differences were found between the two groups in any of the health-related quality of life domains studied. Nevertheless, the trial revealed a positive impact of PFM training on the PFM function.

In another study by Manley et al. [12], pelvic floor strength was investigated prior to and post robot-assisted laparoscopic radical prostatectomy. The exercise used included coordination, reflex action, strength and endurance training. Pelvic floor muscle strength four weeks after catheter removal was associated with continence. The only significant predictor of incontinence was advanced age. The lack of a control group, subjective assessment of pelvic muscle strength and some loss to follow-up limited the evaluation of the outcomes. However, the authors positively assessed preoperative pelvic floor muscle training as improving surgical outcomes for men undergoing robot-assisted laparoscopic radical prostatectomy. This conclusion was confirmed in a meta-analysis including eleven trials with a total number of 739 patients who underwent prostatectomy. The subjects who were allocated to the PFM training groups received before-surgery training sessions that ranged in various studies from 20 min to 1 h in length and from once to twice weekly. The analysis showed a significantly lower rate of postoperative incontinence at three months in the PFMT group compared with the control group (*p* = 0.005). However, no improvement in the long-term continence rate was demonstrated. No significant differences were revealed between the groups at six months in postoperative incontinence (*p* = 0.12) [13].

Another systematic review concerning PFM training preprostatectomy based on nine RCTs revealed a significant improvement in postsurgical urinary incontinence, reduction in erectile dysfunction and postmicturition dribble, regardless of the PFM training regimen [14]. Similar observations were made by Tienforti et al. who, in a prospective randomized study based on 34 participants undergoing open radical prostatectomy, showed a beneficial effect of a postoperative monthly supervised pelvic exercise program, preceded by a preoperative educational session and PFM training with biofeedback, on postoperative urinary incontinence. In the six-month follow-up, men in the intervention group reported faster recovery of voiding control after surgery, lower number of incontinence episodes and pads per week compared to the control group, which received only oral and written instructions on postoperative pelvic floor exercises performed at home [15]. These outcomes were not confirmed by the study reporting the effects of preoperative PFM training with biofeedback on stress urinary incontinence and quality of life in men undergoing laparoscopic radical prostatectomy. The analysis was based on 248 men randomly allotted into one of two groups (PFM training with biofeedback and surgery or surgery alone). The study did not show a beneficial effect of preoperative PFMT on postoperative stress urinary incontinence and quality of life [16]. This observation was confirmed by an RCT conducted on 180 men undergoing radical prostatectomy. The median urinary continence recovery time was similar in the preoperative pelvic floor exercise group and in the group with only postoperative PFMT: 30 and 31 days, respectively (*p* = 0.878) [17]. Similar results were revealed in a meta-analysis of five RCTs concerning urinary incontinence after radical prostatectomy in men with preoperative PFM training. The survey showed no improvement in postoperative urinary incontinence in men with preoperative pelvic floor muscle training in any follow-up period ranging from one month to one year [18]. Other authors found no impact of preoperative rectal electrical stimulation of pelvic floor muscles on urinary continence in patients undergoing radical prostatectomy [19]. Details of the studies investigating PREHAB in male pelvic floor surgery are presented in Table 2.

### 2.3. Preoperative Muscle Function and Surgical Outcomes

There is a paucity of evidence on the beneficial effects of preoperative pelvic floor muscle training on surgical outcomes. Some of the recommendations are based on studies showing a correlation between weak pelvic floor muscles before surgery and the deterioration of their efficiency after surgery. Considering the results of the performed trials, the question could be raised of whether preoperative muscle function allows predicting surgical outcomes. Duarte et al. [20] presented a prospective observational study that included 65 women scheduled for POP surgery. Pelvic floor muscles strength was evaluated with the use of the modified Oxford Grading Scale and manometry (measured with the use of a Peritron perineometer instrument) at two time points: 15 days before and 40 days after surgery. The study did not show any statistically significant differences in maximum voluntary contraction nor its duration pre- and postsurgery; however, after surgery, the average contraction was higher and better muscle performance was determined using the modified Oxford Grading Scale. Moreover, the trial confirmed the initial hypothesis assuming a relationship between the pre- and postoperative severity of pelvic organ prolapse and the attenuation of maximal voluntary contraction as well as the duration of pelvic floor muscle contraction. In the study by Manley et al. [12], increasing age associated with weak pelvic floor muscle strength was a strong predictor of poor surgical outcomes after robot-assisted radical prostatectomy. Vakili et al. [21] investigated the correlation between the strength of the levator ani contraction and the genital hiatus measurement with surgical failure in prolapse. The retrospective trial included 358 women scheduled for prolapse surgery. The Oxford Grading Scale was implemented for the recording of the levator contraction strength. A correlation has been demonstrated between the increased or normal strength of the levator ani contraction and the reduced risk of recurrent pelvic organ prolapse (*p* = 0.005 and *p* = 0.017, respectively). A decreased risk of recurrent incontinence (*p* = 0.010), and a lower rate of recurrent surgery (*p* = 0.13) were related to the increasing strength of the levator ani. Reversely, the inability to contract the levator ani was highly associated with recurrent incontinence (*p* = 0.023). The widening of the genital hiatus above 5 cm was related to an increased incidence of recurrent prolapse (*p* = 0.034). This relationship did not apply to the recurrence of urinary incontinence or the need for additional surgery. On the other hand, the need for additional surgery due to prolapse or urinary incontinence was most strongly correlated with the strength of levator ani contraction (*p* = 0.011). The other retrospective review aimed to establish the link between the strength of the pelvic floor muscles and the recurrence of vaginal prolapse in women who have already received surgery for this reason. Two hundred ninety-nine women who met the inclusion criteria were followed for an average of 143.9 weeks. The patients were divided into “absent,” “weak,” and “good” preoperative pelvic muscle strength based on the modified Oxford Grading Scale. The study showed that the recurrence rate of anterior vaginal wall prolapse was significantly higher in women with absent PFM strength (nondetectable PFM contractions) compared to those with weak or good muscle strength (13.89% vs. 3.48% and 4.05%, respectively (*p* = 0.033)) [22].

### 2.4. Could Surgery Improve PFM Function?

On the other hand, prolapse surgery aimed at restoring the proper anatomical positions of the pelvic organs could theoretically help in the recovery of PFM function. Guan et al. [23] reported significantly improved PFM strength in women who underwent the modified pelvic reconstruction procedure. However, the study design and quality of the presented data limits the strength of this evidence and makes drawing further conclusions difficult. The control group consisted of subjects assigned to the oophorectomy procedure. The change between the baseline and postoperative PFM strength was not compared between groups. What is more, the improved strength of PFM may partially be attributed to the pelvic floor re-education process, since, after the initial preoperative assessment, the subjects already knew how to activate these structures. Other parameters of muscle performance were not reported.

A recently published systematic review evaluating the effect of pelvic organ prolapse and urinary incontinence surgery on PFMs in women showed no statistically significant changes in pelvic floor muscle function from six weeks to six months. The review included 21 trials based on 1063 female participants who received varied surgical pelvic organ prolapse/continence interventions. Thirty-three different methods of PFM assessment were implemented in the studies, including the modified Oxford Grading Scale, vaginal squeeze pressure and anorectal squeeze pressure manometry for the assessment of the strength, digital palpation for the evaluation of PFM endurance, electromyography for the assessment of myoelectrical activity, and transperineal ultrasound or magnetic resonance imaging for the evaluation of PFM morphometry. The study presents several limitations, the most important of which is the poor or moderate quality of included papers. The analyzed studies are characterized by the heterogeneity of participants and surgical interventions, insufficiency in establishing associations between the strength of postoperative muscle contraction and the change in their morphometry, as well as the low rating of the grade of the evidence used. Thus, further well-designed prospective trials are necessary to assess the impact of urogynecological surgery on pelvic floor muscle efficiency, while PFM PREHAB is needed to improve surgical outcomes [24].

## 3. Conclusions

The influence of pelvic floor muscle performance on the outcomes of prolapse and incontinence surgery remains an understudied area. The restoration of deteriorated PFM function constitutes a challenge due to the complexity of the muscles, ligaments, fascia and nerves that make up the pelvic floor. The results of the studies on PREHAB published so far are inconclusive and conflicting. The small sample size and the lack of standardization of PFM exercises are a frequent limitation of these studies. Further well-designed prospective trials are necessary to answer the question of whether therapeutic actions should focus on building muscle strength or rather on enhancing muscle performance, such as an awareness of the contraction and relaxation incorporated in everyday activities.

The mechanism and pathophysiology of stress urinary incontinence and pelvic organ prolapse are different. However, some of the above-mentioned studies investigated them together. Although pelvic muscle training can improve both [25], future research should address them as two distinct conditions in order to look at the possible differences in management and PREHAB results.

It is known that correct preoperative preparation affects the results of surgical treatment. Preoperative factors and appropriate risk assessment are important predictors of the medical and economic impact of postoperative outcomes [26]. From an economical point of view, it is important to understand what measures need to be taken to optimally prepare a patient for pelvic floor reconstructive surgery. To what extent should PREHAB be implemented? Would proper patient education be enough or should extensive rehabilitation with an objective muscle performance measurement be applied? Further research is undoubtedly warranted in this area.

## Figures and Tables

**Table 1:** Details of the studies investigating perioperative physiotherapy in female pelvic floor surgery.

Author	Type of Study	Population	Condition Studied	Intervention	Intervention Delivery	Outcome Measures	Results
Jarvis et al., 2005 [6]	RCT	60 females (30 in the PFM training group; 30 in the control group)	UI and/or POP	- Individual PFM training, 4 sets per day - “The Knack” - Education (voiding, defecation techniques)	Perioperatively, continued 12 weeks after surgery	Assessment 12 weeks after surgery	
						Paper towel test	No statistically significant differences between the groups *p* = 0.150, 95% CI: 11.4, 72.3
						Standardized urinary symptom-specific health and quality of life questionnaire	Intergroup mean difference of 3.8 favoring the PFM training treatment group, *p* = 0.017, 95% CI: 0.7, 6.9.
						48 h urinary frequency/volume diary	Mean difference in diurnal frequency between the groups in favor of the PFM training group, *p* = 0.024 (PFM training group mean reduction 1.5, control group mean reduction 0.4).
						Manometry	Significantly different mean maximum squeeze in the PFM group in comparison to the control group *p* = 0.022, 95% CI: 9.92, 0.81. Improvement in the mean maximum squeeze of 2.7 cm H_2_O in the PFM training group, reduction in the mean maximum squeeze of 1.8 cm H_2_O in the control group
Zhang et al., 2016 [9]	Systematic review	5 studies 591 females (292 in the PFM training group; 299 in the control group)	POP (one trial included patients scheduled for POP and/or UI surgery)	In 2 studies, women received individual PFM training and lifestyle advice in combination with different adjunct therapies (biofeedback, electrical stimulation, vaginal balls) In the other 3 studies, women received individual PFM training and lifestyle advice only In PFM training, the number of contraction repetitions varied between 8 and 12 and the exercise frequency varied between 3 and 4 times per day	Perioperatively, the number of treatment sessions varied from 3 to 8 during the follow-up period	Primary outcomes: assessment 3–24 months after surgery Primary: Prolapse symptoms Prolapse-specific quality of life Secondary: Degree of prolapse Pelvic floor muscle function Urinary outcomes Measures of quality of life Bowel outcomes Activity scales PFM training adherence Adverse events	No improvement in primary or secondary outcomes between women in the PFM training group and control group
Sung et al., 2019 [10]	RCT	480 females (242 in the PFM training group; 238 in the control group)	Stress and urgency UI	- Education on pelvic floor anatomy, bladder function and voiding habits - PFM training (individual, progressive, administered daily) - Bladder training- Strategies to control stress and urgency symptoms	One preoperative (2–4 weeks before) and 5 postoperative sessions through 6 months	Primary: Urogenital Distress Inventory (UDI) change (from baseline) in symptoms at 12 months	Statistically significant improvement in the PFM training group vs. the control (sling-only) group (−13.4 points, 95% CI: −25.9 to −1.0, *p* = 0.04;); however, it did not meet the prespecified threshold for clinical importance.
						Secondary: Subscale scores between groups at 12 months.	
						UDI-stress	Statistically significant improvement in the PFM training group vs. the control (sling-only) group. The model-estimated between-group difference (−6.1 points; 95% CI: −12.1 to −0.2; *p* = 0.04) did not meet the prespecified threshold for clinical importance.
						UDI-irritative	No statistically significant difference in the PFM training group vs. the control (sling-only) group. The model-estimated between-group difference: −5.5 points; 95% CI: −11.5 to 0.6; *p* = 0.08
						Other: 3-day bladder diary	Significantly greater mean reduction in urgency incontinence episodes (−1.1 vs. −0.4 daily episodes; adjusted difference, −0.7; 95% CI: −1.2 to −0.1; *p* = 0.02) and total incontinence (−2.4 vs. −1.4; daily episodes difference, −1.0; 95% CI: −1.7 to −0.2; *p* = 0.009) in the PFM training group vs. the control (sling-only) group
						Incontinence Impact Questionnaire	Significantly greater improvements in the PFM training group vs. the control (sling-only) group, reached the prespecified threshold for clinical importance. Difference in difference, −29.7; 95% CI: −51.9 to −7.4, *p* = 0.009
						Patient Global Impression of Improvement	No statistically significant difference
						Overactive Bladder Treatment	No statistically significant difference
						Satisfaction Questionnaire	No statistically significant difference
						Symptom and Health-Related Quality of Life (questionnaires administered at 3, 6 and 12 months after surgery)	No statistically significant difference

RCT: randomized controlled trial; UI: urinary incontinence; POP: pelvic organ prolapse; PFM: pelvic floor muscles; CI: confidence interval.

**Table 2:** Details of the studies investigating PREHAB in male pelvic floor surgery.

Author	Type of Study	Population	Condition Studied	Intervention	Intervention Delivery	Outcome Measures	Results
Ocampo-Trujillo et al., 2014 [11]	Randomized prospective intervention study	16 males (8 in the PFM training group; 8 in the control group)	Patients undergoing radical prostatectomy	Intensive PFM training including: - Voluntary and selective contractions and relaxations of the levator ani muscles - Addition of audible and visual biofeedback	3 times a day for 4 weeks, 30 days prior to surgery	Measures were taken at the beginning of the intervention and 8 weeks after surgery	
						The pressure assessment of the levator ani contraction by surface electromyography	Greater degree of change in the average pressure of the levator ani muscle contraction (F = 9.188; *p* = 0.010) in the PFM training group vs. the control group.
						Continence assessed by a 24 h pad test	75% of the patients who underwent muscle training did not require guards, compared with 50% in the control group (*p* > 0.05). Similar observations in the use of 1–2 guards (35.7% vs. 12.5%; *p* > 0.05).
						Prostate Cancer Index health questionnaire (UCLA-PCI)	After the training program, the PFM training group scored higher in the physical 52.1 ± 3.6 vs. 48.7 ± 3.6) and mental (48.3 ± 5.1 vs. 49.4 ± 4.6) items of the UCLA-PCI questionnaire vs. the control group. However, these differences were not statistically significant
						Muscle morphometry	The participants from the PFM training group had higher values in the cross-sectional area of the external sphincter muscle fibers of the urethra compared to the control group (1313 ± 1075 μm^2^ vs. 1056 ± 844 μm^2^, F = 5.458, *p* = 0.03). There were no changes in other morphometric characteristics, minor diameter (μm^2^) or percentage of central nuclei
Manley et al., 2016 [12]	Pilot study	98 males	Patients undergoing robot-assisted radical prostatectomy	- Individual PFM training including strength, reflex action, coordination and endurance exercises - Education about anatomy	The initial physiotherapy consultation of a 2 h duration PFM training implemented before and after surgery, practiced daily. Consultation and training implemented preoperatively, not stated when exactly	Perineal pelvic floor muscle assessment anteriorly Digital rectal exam to evaluate the external anal sphincter and puborectalis Real-time transabdominal ultrasound assessment for assessment of PFM strength rated as absent, weak, moderate or strong Assessments repeated postoperatively with the exception of the rectal exam due to possible pain	Absence of the control group limits the conclusions of the beneficial effects of PFM training prior to surgery
Chang et al., 2016 [13]	Systematic review and meta-analysis	11 studies in a systematic review, 7 studies in meta-analysis	Patients undergoing radical prostatectomy	Different PFM training protocols, with or without biofeedback	In the majority of studies, the first session was 2–4 weeks prior to the surgery. Two studies had their first session 1 day before surgery. Some of the studies did not clearly state the beginning of preoperative PFM training. Duration of PFM exercises varied from 20 min to 1 h in length, frequency from twice a week to weekly	Continence rates (different definitions across the studies)	Significantly lower rates of postoperative incontinence at 3 months postsurgery in the PFM training group compared with the control group, with an OR of being incontinent of 0.64 (*p* = 0.005). There was no significant difference in postoperative incontinence rates at 1 month (OR: 0.68; *p* = 0.07) or 6 months (OR: 0.60; *p* = 0.12)
						Quality of life (American Urological Association Symptom Index, King’s Health Questionnaire (KHQ), University of California Los Angeles Prostate Cancer Index (UCLA-PCI), International Consultation on Incontinence Questionnaire (ICIQ), International Prostate Symptom Score (IPSS))	Seven studies measured quality of life. Four studies showed statistically significant improvements in the PFM training group at 3 months postsurgery
Goonewardene et al., 2018 [14]	Narrative review	9 studies	Patients undergoing robotic radical prostatectomy	Different PFM training protocols, with or without biofeedback	Different PFM training delivery	Continence rates, incidence, duration and severity	Statistically significant improvements in the PFM training groups regardless of the PFM training regimen
Tienforti et al., 2012 [15]	A prospective, single-center RCT	34 males (17 in the PFM training group; 17 in the control group)	Patients undergoing standard open retropubic radical prostatectomy	- Supervised training session with biofeedback - Oral and written instructions on pelvic floor muscle contractions. Three sets of 10 min each (5 s contractions then 5 s relaxations) - Education about anatomy and physiology of the lower urinary tract	The day before surgery and immediately after catheter removal, repeated daily Exercise frequency was recorded in a training diary	Outcome assessment performed monthly for the PFM training group and at 1, 3 and 6 months after catheter removal for the control group	
						Primary: Self-reported recovery of continence 6 months after catheter removal (continence defined by the International Consultation on Incontinence Questionnaire on Urinary Incontinence (ICIQ-UI) as a score of zero)	The difference between groups was statistically significant at each reported follow-up time favoring the PFM training group
						Secondary: Number of incontinence episodes per week	The number of incontinence episodes per week was significantly lower for patients in the PFM training group at both the 3 (3.84 vs. 14, *p* = 0.01) and 6-month follow-ups (2.72 vs. 13.06, *p* = 0.005)
						Number of pads used per week	The number of pads per week was significantly lower for patients in the PFM training group at boththe 3 (1.50 vs. 6.25, *p* = 0.005) and 6-month follow-ups (1.31 vs. 4.625, *p* = 0.03)
						Overactive bladder symptoms, measured by the International Consultation on Incontinence Questionnaire Overactive Bladder Module (ICIQ-OAB)	ICIQ-OAB scores showed significant differences in favor of the PFM training group at the 3- (10.12 vs. 13.19, *p* = 0.04) and 6-month follow-ups (9.06 vs. 12.62, *p* = 0.01)
						Urinary function measured by the University of California Los Angeles Prostate Cancer Index (UCLA-PCI)	UCLA-PCI scores showed significant differences in favor of the PFM training group at the 3- (403.81 vs. 272.44, *p* = 0.006) and 6-month follow-ups (422.50 vs. 274.25, *p* = 0.003)
						Impact of incontinence on quality of life measured by the International Prostate Symptom Score (IPSS-QoL)	Patients in the PFM training group reported lower IPSS-QoL scores (better quality of life) than those in the control group at all follow-up times but the difference was not statistically significant
Dijkstra-Eshuis et al., 2015 [16]	RCT	248 males (124 in each group)	Patients undergoing laparoscopic radical prostatectomy	30 min sessions of PFM training with biofeedback (maximal voluntary contractions, endurance, relaxation and coordination with abdominal breathing) - Education about toilet behavior	Once weekly, four weeks prior to surgery	Assessments at 6 weeks, 3 months, 6 months, 9 months and 1 year postoperatively King’s Health Questionnaire (KHQ) International Prostate Symptom Score (IPSS) 24 h bladder diary 24 h pad test	There were no significant differences between the PFM training group and the control group in terms of the incidence of urinary incontinence and quality of life measured by KHQ and IPSS 6 weeks, 3, 6 and 9 months and 1 year postoperatively (*p* > 0.05)
Geraerts et al., 2013 [17]		180 males (91 in the PFM training group; 89 in the control group)	Patients undergoing open radical prostatectomy and robot-assisted laparoscopic radical prostatectomy	- Individual PFM training program (exercises of the pelvic floor manually controlled by the therapist and electromyography biofeedback once a week). Additionally, patients performed a home program of 60 contractions per day - Education on contracting the pelvic floor muscles while coughing and sitting down or getting up from a chair	3 weeks before surgery and continued after surgery. Supervised 30 min sessions once a week and daily home exercises The control group started PFM training after catheter removal	Assessment before surgery and 1, 3, 6 and 12 months after surgery	
						Primary: Time to continence (24 h pad test)	Time to continence comparable between PFM training and control groups during the first year after surgery (*p* = 0.878). Compared with controls, patients in the PFM training group had comparable cumulative incidence rates for continence and average amount of urine loss at all time points
						Secondary: The point prevalence of urinary continence (0 or 1 g on the 1 h pad test and the Visual Analogue Scale (VAS))	Comparable for both groups at 1, 3, 6 and 12 months after surgery
						International Prostate Symptom Score (IPSS)	Did not differ between the groups at any time point.
						King’s Health Questionnaire (KHQ)	Only one aspect of the KHQ, incontinence impact, was in favor of the PFM training group at 3 (*p* = 0.008) and 6 months after surgery (*p* = 0.024)
Wang et al., 2014 [18]	Meta-analysis	5 studies	Patients undergoing radical prostatectomy	Of the five, two trials implemented PFM training with biofeedback, three trials used physiotherapist-supervised PFM training	PFM training started 2–4 weeks before surgery	Urinary continence at different time points (1, 3, 6 and 12 months after surgery)	PFM training before surgery did not improve the reestablishment of urinary continence after radical prostatectomy
						Time to continence	Narrative analysis: no significant difference between groups in included studies
						Quality of life	Narrative analysis: inconsistent results about differences in quality of life between the groups in included studies
Laurienzo et al., 2013 [19]	RCT	49 males (3 randomized groups: 15 in the control group, 17 in the exercise group and 17 in the electrical stimulation group)	Patients undergoing radical retropubic prostatectomy	The electrical stimulation group: 10 physiotherapy sessions before surgery, using electrical stimulation and rectal pelvic exercises (5 types) The exercise group: 10 physiotherapy sessions before surgery, only the pelvic exercises. The exercises were the same as in the electrical stimulation group	Variable frequency (respecting scheduled surgery)	Assessment 1, 3 and 6 months after the surgical procedure	
						1 h pad test	No significant difference between the 3 groups at 1, 3 and 6 months of follow-up (*p* > 0.05). Based on the odds ratios between groups, there was no significant difference (*p* > 0.05), with a 95% confidence interval.
						International Consultation on Incontinence Questionnaire-Urinary Incontinence Short Form (ICIQ-UI SF)	No significant difference in ICIQ-UI SF score between the 3 groups at 1, 3 and 6 months of follow-up (*p* > 0.05)
						Short Form Health Survey (SF-36)	No differences between groups on the various domains of the SF-36 (*p* > 0.05)

PREHAB: Prehabilitation; RCT: randomized controlled trial; PFM: pelvic floor muscles; OR: Odds ratio.
